# Comparison and validation of the prognostic value of preoperative systemic immune cells in hepatocellular carcinoma after curative hepatectomy

**DOI:** 10.1002/cam4.1424

**Published:** 2018-03-13

**Authors:** Rui Liao, Cong Peng, Ming Li, De‐Wei Li, Ning Jiang, Pei‐Zhi Li, Xiong Ding, Qiao Wu, Cheng‐You Du, Jian‐Ping Gong

**Affiliations:** ^1^ Department of Hepatobiliary Surgery The First Affiliated Hospital of Chongqing Medical University Chongqing 400016 China; ^2^ Department of Pathology Chongqing Medical University Chongqing 400016 China; ^3^ Department of Hepatobiliary Surgery The Second Affiliated Hospital of Chongqing Medical University Chongqing 400010 China; ^4^ Chongqing Key Laboratory of Hepatobiliary Surgery The Second Affiliated Hospital of Chongqing Medical University Chongqing 400010 China

**Keywords:** Inflammation, liver cancer, nomogram, prognosis, surgery

## Abstract

In this study, we aimed to compare and validate the prognostic abilities of preoperative systemic immune cells in hepatocellular carcinoma (HCC) after curative hepatectomy. We developed two nomograms to predict the postoperative recurrence‐free survival (RFS) and overall survival (OS) after comparisons of the systemic immune cell prognostic scores. The two nomograms were constructed based on 305 patients who underwent curative hepatectomy for HCC. The predictive accuracy and discriminative ability of the nomograms were compared with six commonly used staging systems for HCC. The results were validated using bootstrap resampling and an internal validation cohort of 142 patients and an external validation cohort of 169 patients. Necroinflammatory activity in peritumoral liver tissues in the primary cohort was evaluated by hematoxylin and eosin (H&E) staining. Neutrophil, monocyte, and lymphocyte ratio (NMLR) had a higher area under the receiver operating characteristic curves (AUROC) value at both RFS (AUC = 0.603) and OS (AUC = 0.726) compared to that of other systemic immune cell prognostic scores. The independent predictors of RFS or OS, including *α*‐fetoprotein (AFP), tumor size, tumor number, microvascular invasion, and NMLR, were incorporated into the two nomograms. In the primary cohort, the C‐indexes of the RFS and OS nomograms were 0.705 and 0.797, respectively. The ROC analyses showed that the two nomograms had larger AUC values (0.664 for RFS and 0.821 for OS) than those of the American Joint Commission on Cancer seventh edition, Barcelona Clinic Liver Cancer, Cancer of the Liver Italian Program, Japan Integrated Staging Score, Okuda stage, and the Vauthey's system. These results were verified by internal and external validations. The nomogram‐predicted probability of RFS was associated with peritumoral necroinflammatory activity scores (*r* = 0.304, *P* < 0.001). The proposed nomograms had accurate prognostic prediction in patients with HCC after curative hepatectomy.

## Introduction

Hepatocellular carcinoma (HCC) is the third most lethal malignancy and has a high incidence among all human neoplasms worldwide 1. However, although curative partial liver resection offers the chance of long‐term survival for patients with HCC, the 5‐year recurrence rate remains approximately 50–75% 2, 3. Therefore, it is reasonable to stratify at‐risk subpopulations for the purposes of individualizing surveillance and optimizing postoperative rational treatment without delay.

In contrast to other cancers, the prognosis of HCC is not solely dependent on the tumor progression but also on adverse impacts from the clinical consequence of cirrhosis 4. A myriad of pro‐inflammatory stimuli, such as hepatitis B or C viral (HBV or HCV) infection, are accumulated around the inflamed liver parenchyma and initiate and sustain the process of HCC and trigger relapse 5. It is clear that crosstalk between cancer cells and their surrounding hepatic immune cells is required for tumor phenotype change, angiogenesis, proliferation, and increased invasion abilities 6, 7, 8. For example, lymphocytes act as a double‐edged sword in hepatocarcinogenesis because they exert pro‐ (CD4^+^ Treg lymphocytes) and antitumor effects (natural killer cells and CD8^+^ T lymphocytes) 9. In HCC microenvironments, tumor‐activated neutrophils and monocytes can polarize T‐cell responses and redirect inflammatory responses into tumor development 10. Cancer can stimulate platelet activity; conversely, platelets can interact with tumor cells and produce several growth factors that facilitate cancer growth, migration, epithelial–mesenchymal transition, and metastasis 11, 12. Our recent studies also supported the predictive roles of systemic immune cells (peripheral neutrophil, monocyte, and lymphocyte ratio, neutrophil‐to‐lymphocyte ratio (NLR), monocyte‐to‐lymphocyte ratio (MLR), and neutrophil, monocyte, and lymphocyte ratio (NMLR)) in HCC 13, 14. Similarly, a number of studies have shown that preoperative peripheral immune cell counts or ratios such as monocyte counts 15, platelet–lymphocyte ratio (PLR) 16, and platelet–neutrophil–lymphocyte ratio (PNLR) 17 were potential risk factors for dropout before and for recurrence of patients with HCC after liver resection. However, the count or ratio that is most suitable for predicting outcome in patients with HCC remains to be determined.

As an alternative or better predictive model compared to the traditional staging systems, several nomograms have recently been developed for HCC 3, 18, 19. To the best of our knowledge, there is no specific nomogram for resected HCC prognosis incorporating systemic immune cell counts or ratios based on a comprehensive comparison analysis of their prognostic abilities that could reflect the balance of host inflammatory and immune status. Therefore, in this study, we aim to compare the prognostic performance of preoperative peripheral immune cells and develop reliable nomograms providing accurate estimations of the prognosis of HCC that might be due to long‐term inflammation impact.

## Materials and Methods

### Patients and study design

We accrued data from the First Affiliated Hospital of Chongqing Medical University between January 2002 and December 2012. A total of 501 consecutive patients undergoing liver resection with curative intent for pathologically confirmed HCC were considered for this retrospective study. Fifty‐four patients were excluded according to the inclusion and exclusion criteria as described previously 4. Finally, 447 patients qualified for this study and were further divided into a primary cohort (January 2002 to December 2009, *n* = 305) to develop the nomograms and an internal validation cohort (January 2010 to December 2012, *n* = 142).

To serve as an external validation cohort, we used another independent cohort of 169 consecutive patients with histologically proven HCC after surgery selected from the Second Affiliated Hospital of Chongqing Medical University between January 2007 and July 2011.

This study was performed in compliance with the 1975 Helsinki Declaration and was specifically approved by the Ethics Review Committee of the First Affiliated Hospital of Chongqing Medical University and the Second Affiliated Hospital of Chongqing Medical University. The Reporting Recommendation for Tumor Marker (REMARK) guidelines were used to conduct and report this study. Written informed consents were provided for patients before surgery and were obtained from all patients.

### Follow‐up and treatment for tumor recurrences

After discharge from the hospitals, all patients underwent follow‐up once every month for the first year and subsequently every 3 months from the 7th to 24th months and then every 6 months thereafter. According to the postoperative time, the follow‐up program at each of the visits included serum *α*‐fetoprotein (AFP), serum biochemistry, abdomen ultrasonography, chest X‐ray and abdominal computed tomography (CT), or/and magnetic resonance imaging (MRI) examination. Conventional tumor‐related variables comprising tumor size, tumor capsule formation, microvascular invasion, and Barcelona Clinic Liver Cancer (BCLC) stage were recorded and assessed as described previously 4.

Patients with recurrence received further treatment including a second liver resection, radiofrequency ablation, percutaneous ethanol injection, transcatheter arterial chemoembolization, or symptomatic treatment. The treatment strategies for tumor recurrences depend on the tumor size, site, number, liver functional reserve, extent of disease, and general health of the patient. The RFS and OS were defined as the interval between the date of surgery and recurrence or date of patient death or last follow‐up, respectively. Recurrence was subdivided into early (≤24 months) and late recurrence (>24 months).

### Systemic immune cell prognostic scores

Systemic immune cell prognostic scores including NLR, MLR, PLR, PNLR, PMLR (platelet counts x monocyte counts/lymphocyte counts), and NMLR were constructed. X‐Tile software (Yale University, New Haven, CT) was used to estimate an optimal cutoff of the prognostic scores values based on tumor recurrence in the primary cohort, according to our previous report 13.

### Tissue microarray and evaluation of necroinflammatory activity

A tissue microarray (TMA) was constructed as described previously 13. Triplicate cores of 1 mm were taken from each formalin‐fixed, paraffin‐embedded surgical specimen from the non‐necrotic tissue. The peritumoral tissue was 1.5 cm from the border of HCC tissues. Serial surgical specimens were stained by hematoxylin and eosin (H&E) and assessed by two experienced hepatopathologists. The degree of necroinflammatory activity in the liver tissue was evaluated as described by Ishak et al. 20. The assessment of necroinflammatory activity was based on the extent and distribution of the predominantly inflammatory infiltrate characteristics including portal, periportal, and intra‐acinar inflammatory cell infiltration, and liver cell necrosis. Briefly, necroinflammatory activity in the liver tissues was divided into the following four levels: Grade 1 (1–4): no activity; Grade 2 (5–8): mild; Grade 3 (9–12): moderate; and Grade 4 (13–18): severe 20, 21.

### Statistical analysis

All statistical analyses were completed with SPSS 16.0 for Windows (SPSS, Inc., Chicago, IL). Categorical variables were compared using the chi‐square test or Fisher's exact test. Continuous variables were compared using Student's t‐test or nonparametric Mann–Whitney *U*‐tests. The correlation between variables was analyzed with Pearson's or Spearman's *ρ* coefficients tests. The sensitivity and specificity were defined by applying receiver operating characteristic (ROC) curves. Survival curves were calculated by Kaplan–Meier survival estimates and compared using the log‐rank test. Factors found to be significant were then included in multivariate analyses using the multivariate Cox proportional hazard regression model to estimate the RFS and OS.

Two nomograms were built based on the results of the multivariable analyses of RFS and OS in the primary cohort using the package of rms in R version 3.4.0 (http://www.r-project.org/). A backward step‐down selection process was performed for the final model selection according to the Akaike information criterion 22. Discrimination was evaluated by calculating the concordance index (C‐index). Calibration was evaluated using calibration plots, which compared the predicted survival by the Kaplan–Meier curves of the quartiles of predictions. The C‐index and calibration curve were derived based on the regression analysis. The values of the C‐index range from 0.5 (no discrimination) to 1.0 (perfect discrimination) 23. Bootstraps with 1000 resample were used for both the validation of the nomograms and for calibration assessment. Bootstraps were also used to correct the regression coefficients, the C‐index, and the variance for overoptimism explanation. All statistical tests were two‐tailed, and a *P* value <0.05 was considered statistically significant. On the basis of the ROC curve analysis, we compared the prognostic nomograms with six conventional clinical staging systems including the American Joint Commission on Cancer (AJCC) seventh edition 24, BCLC 25, Cancer of the Liver Italian Program (CLIP) 26, Japan Integrated Staging Score (JIS) 27, Okuda stage 28, and the Vauthey's system 29.

## Results

### Baseline characteristics

The baseline characteristics of the 616 patients with HCC in the primary and validation cohorts are described in Table 1. The median follow‐up time was 42.5, 40.5, and 41.0 months. Fifty‐four patients were excluded because of the presence of preoperative extrahepatic metastases, preoperative anticancer treatments, incomplete patient records, or lost to follow‐up. The median age was 52, 51, and 53 years in the primary, internal validation, and external validation cohorts, respectively. In the primary cohort, the majority of patients were hepatitis B surface antigen (HBsAg) positive (82.6%) and had a single tumor (86.9%); 96 (31.5%) patients had microvascular invasion. The median tumor size was 4.0 cm. Similarly, in the internal validation and external validation cohorts, most patients were HBsAg positive (89.4% and 82.2%, respectively) and had a single tumor (90.8% and 87.0%, respectively). Microvascular invasion occurred in 26.8% and 24.9% patients, and the median tumor sizes were 4.0 and 3.7 cm, respectively. Of the 616 patients in the three cohorts, the neutrophil, monocyte, and lymphocyte counts ranged from (0.5–14.0) × 10^9^/L, (0.1–1.4) × 10^9^/L, and (0.1–11.0) × 10^9^/L, respectively. The baseline clinicopathologic characteristics were broadly similar among these three cohorts (all *P* > 0.05).

**Table 1:** Characteristics of patients in the primary and validation cohorts

Characteristics	Primary cohort *n* = 305	Internal validation cohort *n* = 142	External validation cohort *n* = 169	*P*‐value
Age, year, median, (range)	52 (18–79)	51 (25–78)	53 (18–78)	0.439
Gender (Female/Male)	48/257 (15.7%/84.3%)	20/122 (14.1%/85.9%)	24/145 (14.2%/85.8%)	0.858
Cirrhosis (yes/no)	266/39 (87.2%/12.8%)	134/8 (94.4%/5.6%)	149/20 (88.2%/11.8%)	0.069
Laboratory test				
ALT U/L, median (range)	39.0 (8.0–949.0)	39.0 (15.0–370.0)	43.0 (8.0–835.0)	0.667
AST, U/L, median (range)	37.0 (12.0–587.0)	33.0 (11.0–334.0)	36.0 (11–527.0)	0.396
GGT, U/L, median (range)	53.0 (7.0–693.0)	61.0 (13.0–513.0)	57.0 (7.0–388.0)	0.913
ALB, g/L, median (range)	44.0 (30.0–60.0)	43.0 (31.0–54.0)	43.0 (33.0–55.0)	0.235
TBIL, *μ*mol/L, median (range)	14.0 (5.9–146.6)	10.8 (4.5–34.4)	13.2 (4.7–95.3)	0.712
AFP, ng/mL, median (range)	74.0 (0–60500.0)	125.5 (0–60500.0)	31.0 (0–60500)	0.367
Neutrophil, 10^9^/L, median (range)	3.5 (0.5–14.0)	2.9 (0.4–12.6)	3.4 (0.9–9.5)	0.176
Monocyte, 10^9^/L, median (range)	0.3 (0.1–1.4)	0.4 (0.1–0.9)	0.4 (0.1–1.1)	0.438
Lymphocyte, 10^9^/L, median (range)	1.7 (0.1–11.0)	1.6 (0.3–3.5)	1.6 (0.3–3.0)	0.913
Platelet, 10^9^/L, median (range)	140.0 (30.0–468.0)	145.0 (39.0–356.0)	145.0 (46.0–333.0)	0.938
HBsAg (Positive/Negative)	252/53 (82.6%/17.4%)	127/15 (89.4%/10.6%)	139/30 (82.2%/17.8%)	0.139
Tumor characteristics				
Tumor number (single/multiple)	265/40 (86.9%/13.1%)	129/13 (90.8%/9.2%)	147/22 (87.0%/13.0%)	0.455
Vascular invasion (yes/no)	96/209 (31.5%/68.5%)	38/104 (26.8%/73.2%)	42/127 (24.9%/75.1%)	0.268
Tumor capsule (complete/Incomplete)	166/139 (54.4%/45.6%)	72/70 (50.7%/49.3%)	92/77 (54.4%/45.6%)	0.737
Tumor differentiation (I–II/III–IV)	230/75 (75.4%/24.6%)	103/39 (72.5%/27.5%)	124/45 (73.4%/26.6%)	0.779
Tumor size, cm, median (range)	4.0 (1.0–20.0)	4.0 (1.5–21.0)	3.7 (1.2–18.0)	0.680
Cancer staging system				
BCLC stage (0–A/B–C)	143/162 (46.7%/53.3%)	78/64 (54.9%/45.1%)	89/80 (52.7%/47.3%)	0.221
AJCC stage (I/II–III)	211/94 (69.2%/30.8%)	95/47 (66.9%/30.1%)	119/50 (70.4%29.6%)	0.797
Okuda stage (I/II)	284/21 (93.1%/6.9%)	132/10 (93.0%/7.0%)	158/11 (93.5%/6.5%)	0.981
Vauthey stage (I/II–III)	140/165 (45.9%/54.1%)	68/74 (47.9%/52.1%)	83/86 (49.1%/50.9%)	0.786
CLIP score (0–1/2–4)	237/68 (77.7%/22.3%)	115/27 (81.0%/19.0%)	129/40 (76.3%/23.7%)	0.598
JIS score (1–2/3–4)	189/116 (62.0%/38.0%)	100/42 (70.4%/29.6%)	120/49 (71.0%/29.0%)	0.070

AFP, alpha fetoprotein; AJCC, American Joint Committee on Cancer; ALB, albumin; ALT, alanine aminotransferase; AST, aspartate aminotransferase; BCLC, Barcelona Clinic Liver Cancer; CLIP, The Cancer of the Liver Italian Program; GGT, gamma‐glutamyl transpeptidase; HBsAg, hepatitis B virus surface antigen; JIS, Japan Integrated Staging; TBIL, total bilirubin.

### RFS and OS in the three cohorts

For the primary cohort, the median RFS was 34.0 months (range, 1.0–82.0 months). The 1‐, 3‐, and 5‐year RFS rates were 71.6%, 54.2%, and 30.4%, respectively. The median OS was 36.0 months (range, 1.0–82.2 months), and the 1‐, 3‐, and 5‐year OS rates were 83.5%, 68.8%, and 49.1%, respectively.For the internal validation and external validation cohorts, the median RFS were 33.4 months (range, 1.0–55.0 months) and 35.5 months (1.0–79.3 months), respectively. The 1‐ and 3‐year RFS rates were 67.0% and 44.0%, and 70.5% and 46.2%, respectively.The median OS was 39.1 months (range, 2.0–55.0 months) and 36.1 months (range, 1.5–81.0 months), respectively. The 1‐ and 3‐year OS rates were 81.9% and 71.4%, and 84.9% and 70.9%, respectively.

### Predictive accuracy comparison of the systemic inflammatory/immune cell prognostic scores in the primary cohort

**Figure 1:**
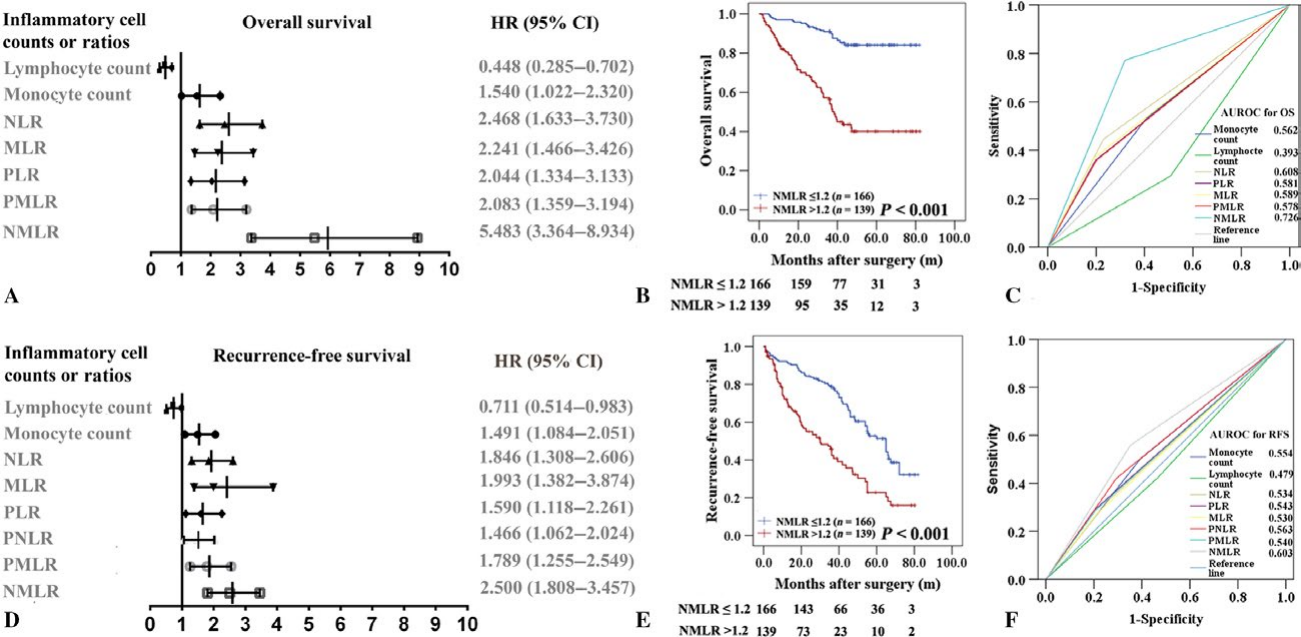
Predictive accuracy comparison of the systemic immune cells prognostic scores for overall survival (OS, A‐C) and recurrence‐free survival (RFS, D‐F) in the primary cohort. Peripheral neutrophil, monocyte, and lymphocyte ratio (NMLR) could predict OS (B) and RFS (E) of patients with HCC after surgery. The hazard ratio (HR) and confidence interval (CI) of OS (A) and RFS (D) rates were analyzed using the Kaplan–Meier method for the systemic immune cells counts and ratios. ROC curves were used to compare the predictive accuracy of the systemic immune cells prognostic scores for assessing OS (C) and RFS (F) rates.

Before assessing the discrimination ability of each scoring system, the relationships between the prognostic scores and the RFS or OS rates were analyzed using the Kaplan–Meier method and compared using log‐rank tests. As shown in Figure 1A and D and Table S1, lymphocyte counts (*P* = 0.037 and <0.001), monocyte counts (*P* = 0.013 and 0.037), NLR (both *P* < 0.001), MLR (both *P* < 0.001), PLR (*P* = 0.005 and 0.001), PMLR (both *P* = 0.001), and NMLR (Fig. 1B and E, both *P* < 0.001) were associated with both the RFS and OS. The PNLR (*P* = 0.019) could predict RFS but not OS. Then, ROC curves were constructed, and the area under the curve (AUC) was used to compare the discrimination ability of each scoring system (Fig. 1C and F). NMLR consistently had a higher AUC value for both the RFS (AUC = 0.603) and OS (AUC = 0.726) compared with the other prognostic scores.

### Independent prognostic factors and development of RFS and OS nomograms in the primary cohort

**Figure 2:**
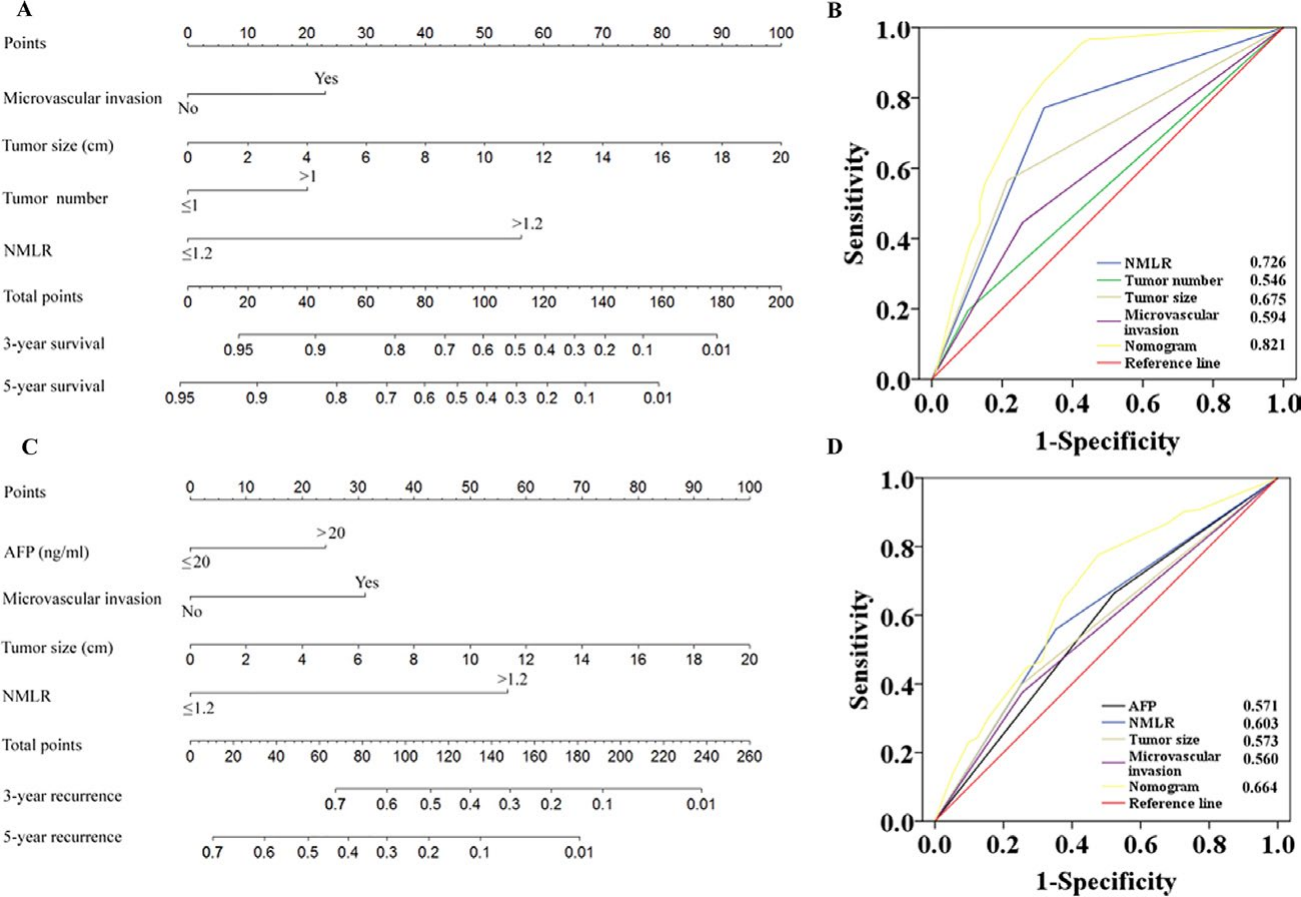
The 3‐ and 5‐year overall survival (OS, A) and recurrence‐free survival (RFS, C) nomograms and predictive accuracy comparison of each variable included in the OS (B) and RFS (D) nomograms by ROC curve analyses. For each predictor, a straight upward line is drawn to determine the points accrued. The sum of these points is plotted on the total points bar, and a straight downward line yields the 3‐ and 5‐year survival rates. The ROC curves showed that the two nomograms were superior to the other variables in predictive accuracy.

After the univariate analyses of our data (Table S1), multivariate analyses were performed on significant clinical factors and demonstrated that vascular invasion (*P* = 0.002 and <0.001), tumor size (*P* = 0.003 and <0.001), and NMLR (both *P* < 0.001) were independent prognostic factors of the RFS and OS. Moreover, AFP was related to the RFS (*P* = 0.035). Monocyte counts (*P* = 0.002) and tumor number (*P* = 0.017) were additional independent predictors for the OS, respectively (Table 2). Furthermore, the independent risk factors of the RFS and OS were incorporated into the RFS and OS nomograms, respectively (Fig. 2A and C, respectively). Monocyte count was not incorporated into OS nomogram that already included the calculation of monocyte count in NMLR which had a higher AUC value for OS.

**Table 2:** Multivariate analysis of overall survival and recurrence‐free survival of HCC in primary cohort

Prognostic variables	OS		RFS	
	HR (95% CI)	*P*‐value	HR (95% CI)	*P*‐value
AFP (>20/≤20 ng/mL)	–	NS	1.447 (1.027–2.040)	0.035
Monocyte (>0.4/≤0.4 × 10^9^/L)	0.473 (0.295–0.758)	0.002	–	NS
Tumor number	1.918 (1.121–3.282)	0.017	–	NS
Vascular invasion (yes/no)	2.136 (1.404–3.249)	<0.001	1.711 (1.221–2.398)	0.002
Tumor size (>5.0/≤5.0 cm)	2.997 (1.966–4.569)	<0.001	1.664 (1.190–2.329)	0.003
NMLR (>1.2/≤1.2)	7.586 (4.328–13.296)	<0.001	2.219 (1.590–3.099)	<0.001

Multivariate analysis: Cox proportional hazards regression model. AFP, alpha fetoprotein; ALB, albumin; CI, confidence interval; HCC, hepatocellular carcinoma; HR, hazard ratio; NS, not significance; OS, overall survival; RFS, recurrence‐free survival.

### Predictive performance of the nomograms in the primary cohort

**Figure 3:**
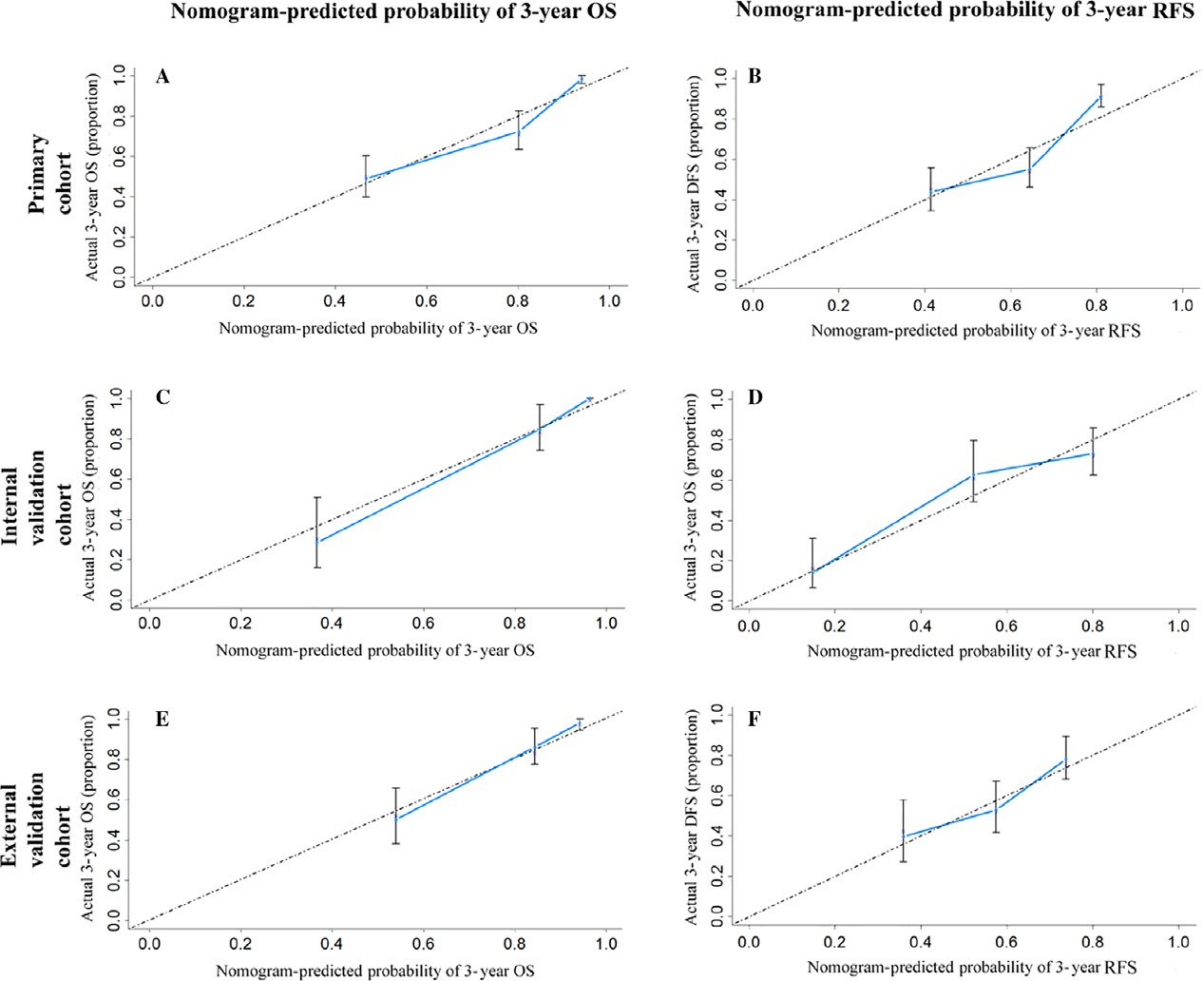
The calibration curves for predicting the 3‐year overall survival (OS, A, C, and E) and recurrence‐free survival (RFS, B, D, and F) rates by nomogram prediction and actual observation in patients with hepatocellular carcinoma in the primary (A and B), internal validation (C and D), and external validation cohorts (E and F).

Among the independent risk factors, the hazard ratios of NMLR for the RFS and OS were higher than the hazard ratios for the other factors (Table 2). The C‐index of the RFS and OS nomograms was 0.705 (95% CI: 0.667–0.743) and 0.797 (95% CI: 0.758–0.836), respectively, which was higher than that of NMLR (C‐index: 0.633 and 0.698) (Table S2). Similarly, by ROC analyses, the RFS and OS nomograms showed the largest AUC (0.664 for RFS and 0.821 for OS, respectively) (Fig. 2B and D) compared with any other independent risk factors incorporated into the nomograms. The calibration plot for the probability of 3‐ or 5‐year RFS and OS after surgery had an optimal agreement between the two nomograms for probabilities and actual observation in the primary cohort, respectively (Figs 3 and S1).

The two nomograms were able to stratify all patients (*n* = 616) into quartiles with significant differences in the RFS and OS rates (5‐year RFS and OS: 51.0% and 96.5% in quartile 1; 27% and 74.7% in quartile 2; 18.8% and 53.1% in quartile 3; and 11.6% and 24.2% in quartile 4, respectively, both *P* < 0.001) (Fig. S2A and B).

The six conventional clinical staging systems all could predict the outcomes of all patients with HCC after surgery (*n* = 616, Figs S3 and S4). As shown in Table S2, the C‐index of the RFS and OS nomograms was significantly higher than that of the AJCC seventh edition stage (0.577 and 0.622), BCLC stage (0.626 and 0.670), CLIP score (0.657 and 0.695), JIS score (0.574 and 0.637), Okuda stage (0.539 and 0.540), and Vauthey stage (0.635 and 0.694). Furthermore, by ROC analyses, the RFS and OS nomograms showed the largest AUC (0.655 for RFS and 0.805 for OS) (Fig. S2C and D) compared to the six conventional clinical staging systems. The results suggest that the two nomograms were accurate predictors for the RFS and OS of patients with HCC after curative hepatectomy.

### Validation of the nomograms

In the internal and the external validation cohorts, the C‐index of the nomograms for predicting the RFS was 0.750 (95% CI: 0.694–0.806) and 0.658 (95% CI: 0.596–0.720), respectively. The C‐indexes of the OS nomogram were 0.887 (95% CI: 0.840–0.934) and 0.781 (95% CI: 0.722–0.840). Both calibration curves of the two nomograms had good agreement between predictions and observations in the probability of 3‐ and 4‐ or 5‐year recurrence and survival (Figs 3 and S1). The ROC analyses showed the two nomograms had larger AUCs than any other independent risk factors and than the six clinical staging systems mentioned above (Fig. S5).

### Predictive performance of the nomograms in patients with early recurrence

In the total of 616 patients with HCC, the RFS nomogram could predict recurrence very well. The C‐indexes were 0.681 (95% CI: 0.652–0.710). There were 181 patients with early recurrence (ER, ≤24 months) in the three cohorts, with 84, 48, and 49 patients in the primary, internal validation, and external validation cohorts, respectively. Of the 181 patients with ER, the proposed nomogram also performed well in the OS prediction. The C‐index was 0.678 (95% CI: 0.629–0.727). The calibration curves for the probability of RFS (*n* = 616) and OS (*n* = 181 with ER) at 1 and 2 years also fit well and suggested that these nomograms could be applied for the prediction of patients with HCC with ER (Fig. S6).

### The association between RFS and the peritumoral necroinflammatory activity in the primary cohort

**Figure 4:**
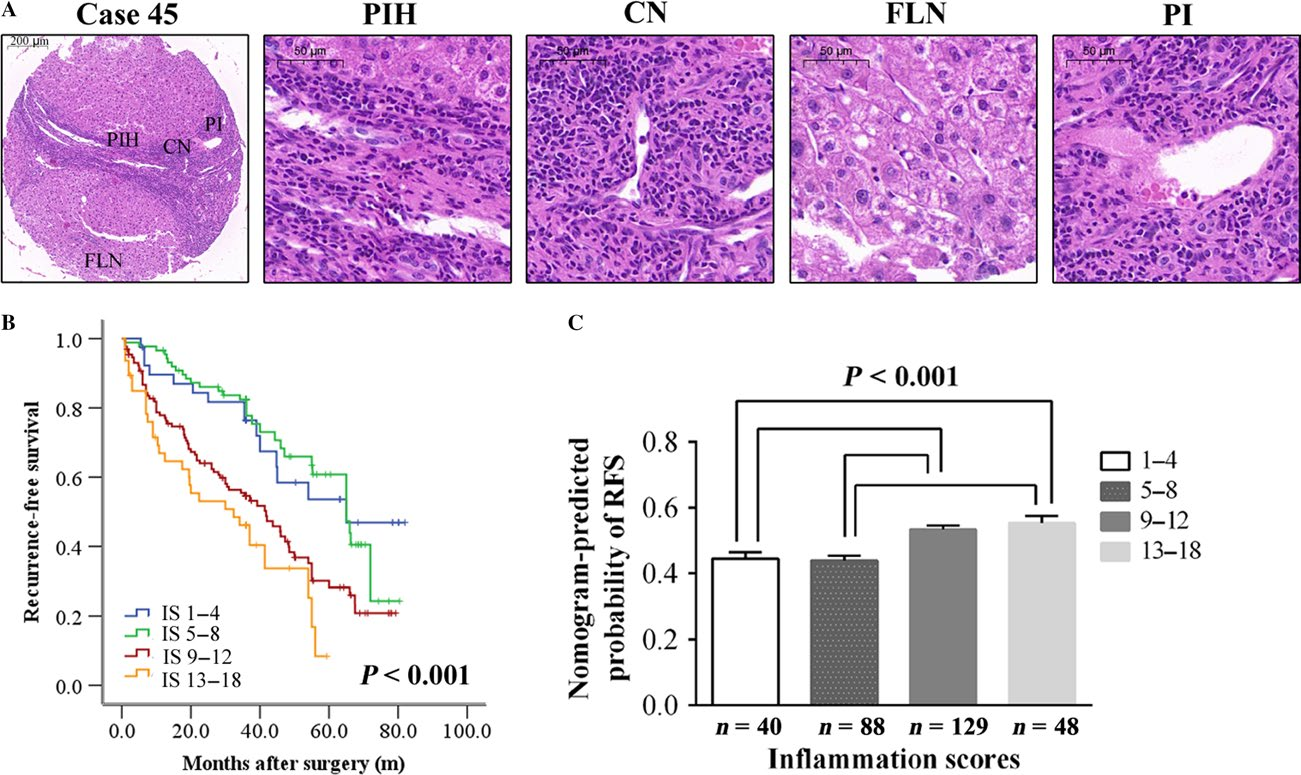
Peritumoral necroinflammatory activity correlates with the recurrence‐free survival (RFS) of patients with HCC. The histomorphology of a typical case (No. 45) showed four inflammation subtypes including periportal or periseptal interface hepatitis (PIH), confluent necrosis (CN), focal (spotty) lytic necrosis and inflammation (FLN), and portal inflammation (PI) by H&E staining (A). Inflammation scores (IS) divided into four grades were associated with the RFS of patients with HCC (*P* < 0.001, B). Compared to low IS (Grade 1–2), high IS (Grade 3–4) was prone to recurrence according to the RFS nomogram prediction (*P* < 0.001, C).

Early recurrence presents as intrahepatic metastases that were remote from the resection line and is closely related to the peritumoral inflammatory environment 30, 31. Therefore, we investigated the association between the RFS and peritumoral necroinflammatory activity in 305 consecutive cases in the primary cohort. Peritumoral inflammatory includes four subtypes as follows (Fig. 4A): periportal or periseptal interface hepatitis (PIH), confluent necrosis (CN), focal (spotty) lytic necrosis and inflammation (FLN), and portal inflammation (PI). The range of inflammation scores (IS) was from 2 to 18, and IS divided into four grades was associated with the RFS of patients with HCC (*P* < 0.001, Fig. 4B). After the parameters of patients with different IS were included into RFS nomogram, we found the patients with high peritumoral IS (Grade 3–4) had a higher nomogram‐predicted probability of RFS than those with low peritumoral IS (Grade 1–2, *P* < 0.001, Fig. 4C). The nomogram‐predicted probability of RFS was associated with peritumoral necroinflammatory activity (*r* = 0.304, *P* < 0.001).

## Discussion

The persistent inflammation activity during the process of HCC formation is complicated and is considered the most important risk factor for tumor prognosis. In particular, the dissemination of primary HCC tumor cells induced by the inflammatory response could result in ER 32, 33. Many studies 13, 15, 16, 17, 34 have showed that elevated systemic immune cell counts and ratios are associated with a poor prognosis in patients with HCC after surgical resection, and all of these counts and ratios are recognized as independent risk factors for the outcomes of HCC. In this study, we demonstrated that the lymphocyte counts, monocyte counts, NLR, MLR, PLR 16, PNLR, PMLR, and NMLR were associated with the RFS and/or OS by the univariate analyses. However, the multivariate analysis showed that only the NMLR was independently associated with both the RFS and OS. We compared the discrimination abilities of these systemic immune cell prognostic scores mentioned above by ROC analysis and found that the NMLR was superior to the others in terms of predictive accuracy. The inflammation microenvironment during the process of HCC formation is complicated and is not invariable, and it involves “crosstalk” and “cooperation” between various immune cells and tumor cells. Our study also supports this point that most single immune cell counts are not able to predict the survival and recurrence of patients with HCC after surgery. However, the NMLR could reflect the complex interaction and potential synergistic effects between the neutrophils and monocyte/macrophages in the tumor milieu. Importantly, the NMLR demonstrated the balance of neutrophils/monocytes and lymphocytes in the systemic inflammatory response. In most tumors, tumor‐activated macrophages differentiate from circulating monocytes and their acquired physiologies and resulting phenotypes contribute to tumor growth, invasiveness, and migration. Tumor‐activated monocytes/macrophages might induce apoptosis of activated CD8^+^ T cells, which have anticancer activities 13. Additionally, increased T lymphocytes can be inflammatory in certain contexts; NMLR may be associated with an anti‐inflammatory phenotype. These findings might partially explain the potential interaction between the NMLR and the immune cells in the tumor milieu. To the best of our knowledge, this is the first report about the predictive accuracy comparison between various systemic immune cell counts and ratios in patients with HCC.

Although the predictive ability of NMLR was reported previously 13, here, it was not solely the analysis period change in previous study. For the first time, we developed two specific OS and RFS nomograms for resected HCC prognosis incorporating systemic immune cell counts or ratios based on a comprehensive comparison analysis of their prognostic abilities that could reflect the balance of host inflammatory and immune status. These nomograms have better predictive abilities and value than NMLR alone. Of all of the risk factors incorporated into the two nomograms, the preoperative AFP level, tumor number, tumor size, and microvascular invasion have been demonstrated to be associated with the surgical prognosis of HCC 30, 35, 36, 37. Up to now, Although AFP is widely used, it is not an ideal predictor for overall survival of HCC after surgery. 30–40% of HCCs after surgery and two‐thirds of patients with HCC smaller than 4 cm have normal AFP levels 17, 38. Interestingly, recent studies showed that HCC recurrence was accompanied by inconsistency in serum AFP 39 and predicted by a high level of preoperative AFP, which is closely associated with both a decreased immunological function in tumor–host and an increased invasive and metastatic ability of HCC cells, thus explaining the high recurrence rate of HCC after surgery 40. Here, tumor number went into the nomogram for OS but not for RFS because majority of the surgical patients (87.8%, 541/616) in this study have single tumor, but the number of nodules (>3) and presence of microvascular invasion were demonstrated to be associated with RFS 41. Therefore, the two nomograms may be more suitable for surgical patients with HCC. The NMLR included into the RFS and OS nomograms might contribute to a significantly increased predictive accuracy due to the close relationship between tumor development and inflammatory response underlying liver diseases. Although these six staging systems in our study showed the ability to stratify patients after surgery into distinct risk categories, the two nomograms had better predictive accuracy for relapse and survival. In the primary and validation cohorts, the C‐index, the calibration curve, and ROC analysis supported that our nomograms were superior to the six conventional staging systems. Compared with any other independent risk factors incorporated into the nomograms and the traditional staging systems commonly used, the RFS and OS nomograms showed the largest AUC (0.664 for RFS and 0.831 for OS, respectively). Although RFS nomogram is not perfect, we think there are more influencing factors of RFS than OS such as treatments after recurrence and some oncogenes inducing metastasis. The predictive accuracy of RFS nomogram could be increased if these significant risk factors are incorporated. Until now, there is no consensus on the follow‐up strategies for HCC recurrent detection after surgery, the prediction model enables surgical patients to be monitored easily on an individual basis.

In clinical practice, it is challenging to predict ER (≤24 months), which stands for a true metastasis 33. Our model is more powerful (C‐index: 0.705, 0.667–0.743) for predicting recurrence after HCC resection than these six conventional staging systems. The RFS nomogram could effectively predict the recurrence at 1 and 2 years (ER) for all patients in these three cohorts. In the patients with ER, the OS nomogram performed well in the survival prediction. The power of prediction of the two nomograms was supported by the C‐index and the calibration curve. These findings might shed light on an important association between the NMLR and ER of HCC, suggesting that the systemic inflammatory response promotes the dissemination of primary HCC tumor cells 13. Therefore, we believe that our models for estimating individualized outcomes following curative hepatectomy might be of more help to clinicians for thoroughly preparing for potential recurrence if patients maintain an underlying constant systemic inflammatory status. Usually, adjuvant chemotherapy would benefit ER which is true metastasis resulting from tumor dissemination before surgery. Also, immune modulation therapy such as IFN‐*α* might improve the outcome for the patients with ER 32.

Our previous study suggested that the peritumoral liver tissue is indisputably the principal target organ for the recurrence of HCC 42, 43. Notably, intrahepatic micrometastases in the peritumoral inflammatory environment mostly occur as ER after surgery. In the present study, more patients with high peritumoral inflammation scores were identified as having a higher recurrence rate. Therefore, we propose that peritumoral inflammatory cells promoted the development and progression of intrahepatic micrometastases, and the inflamed peritumoral liver tissue might serve as a fertile “soil” for micrometastases if endowed with abundant inflammatory cells. Moreover, the nomogram‐predicted probability of RFS was associated with the peritumoral necroinflammatory activity score (*r* = 0.304, *P* < 0.001). This finding revealed that increased production of focal inflammatory cells might mirror increases in circulating inflammatory cell levels and reflect high tumor burdens, contributing to the occurrence of relapse 13. Therefore, comprehensive systemic treatments remain to be carefully evaluated after the tumor is removed.

There are several limitations in the present study. Although C‐reactive protein (CRP) is not a routine examination in the two hospitals, Oh et al. reported 44 that CRP had a significant positive intercorrelation with NLR levels in HCC and the combination of these high levels was identified as a risk factor for worse survival. Therefore, the relationship or difference between NMLR and CRP deserves further study. Some of the patients who received antiviral therapy had irregular medication and drug withdrawal without doctors' advice. Hence, the potential influence of antiviral therapy on the outcomes of patients with HCC after surgery requires further study in the future. Additionally, it is necessary to validate these predictive nomograms in patients without surgery after neoadjuvant or adjuvant therapies. The etiology of liver cirrhosis and HCC is quite different in Asia than in other parts of the world. In particular, toxic cirrhosis is much more common, and HBsAg positivity is much lower in western countries. Therefore, the nomograms may not be suitable for in the countries where hepatitis B is not prevalent. Further comparative study in HCC would facilitate better understanding of the predictive value of the two nomograms in patients with HCC of various etiologies.

The two nomograms developed herein could objectively and reliably predict survival and recurrence in patients with HCC after surgery. This information might help us make informed decisions for triaging patients with HCC at risk of ER following surgery. A large‐scale prospective validation study is needed to determine whether this knowledge can be applied widely.

## Conflict of Interest

No potential conflict of interests.

## Supporting information


**Figure S1.** The calibration curves for predicting the 4‐ or 5‐year overall survival (OS, A, C and E) and recurrence‐free survival (RFS, B, D and F) rates by nomogram prediction and actual observation in patients with hepatocellular carcinoma in the primary (A and B), internal validation (C and D) and external validation cohorts (E and F).

**Figure S2.** Kaplan‐Meier survival curves and patients at risk at each year according to the quartiles of the overall survival (OS, A) and recurrence‐free survival (RFS, B) nomograms and predictive accuracy comparison between the OS (C) and RFS (D) nomograms and six conventional clinical staging systems by ROC curve analyses in the primary cohort.

**Figure S3.** Kaplan‐Meier survival curves of overall survival in the primary (A–F), internal validation (G–L) and external validation cohorts (M–R).

**Figure S4.** Kaplan‐Meier survival curves of the recurrence‐free survival in the primary (A–F), internal validation (G–L) and external validation cohorts (M–R).

**Figure S5.** Predictive accuracy comparison of each variable included in the OS (A and E) and RFS (B and F) nomograms and comparison between the OS (C and G) and RFS (D and G) nomograms and six conventional clinical staging systems by ROC curve analyses in the internal validation (A–D) and external validation (E–H) cohorts.

**Figure S6.** The calibration curves for predicting the 1‐ and 2‐year early recurrence (ER, A and B) in the 616 patients with hepatocellular carcinoma and overall survival (OS, C and D) in the population with ER.

**Table S1**. Univariate analysis of overall survival and recurrence‐free survival of HCC in primary cohort.

**Table S2.** The C‐index of the predictors in nomograms and clinical staging systems.
