# Supplementary figures and images for: Comparison and validation of the prognostic value of preoperative systemic immune cells in hepatocellular carcinoma after curative hepatectomy
Source: Cancer Med. 2018 Mar 13;7(4):1170–82. doi: 10.1002/cam4.1424 (PMC5911633; doi:10.1002/cam4.1424)

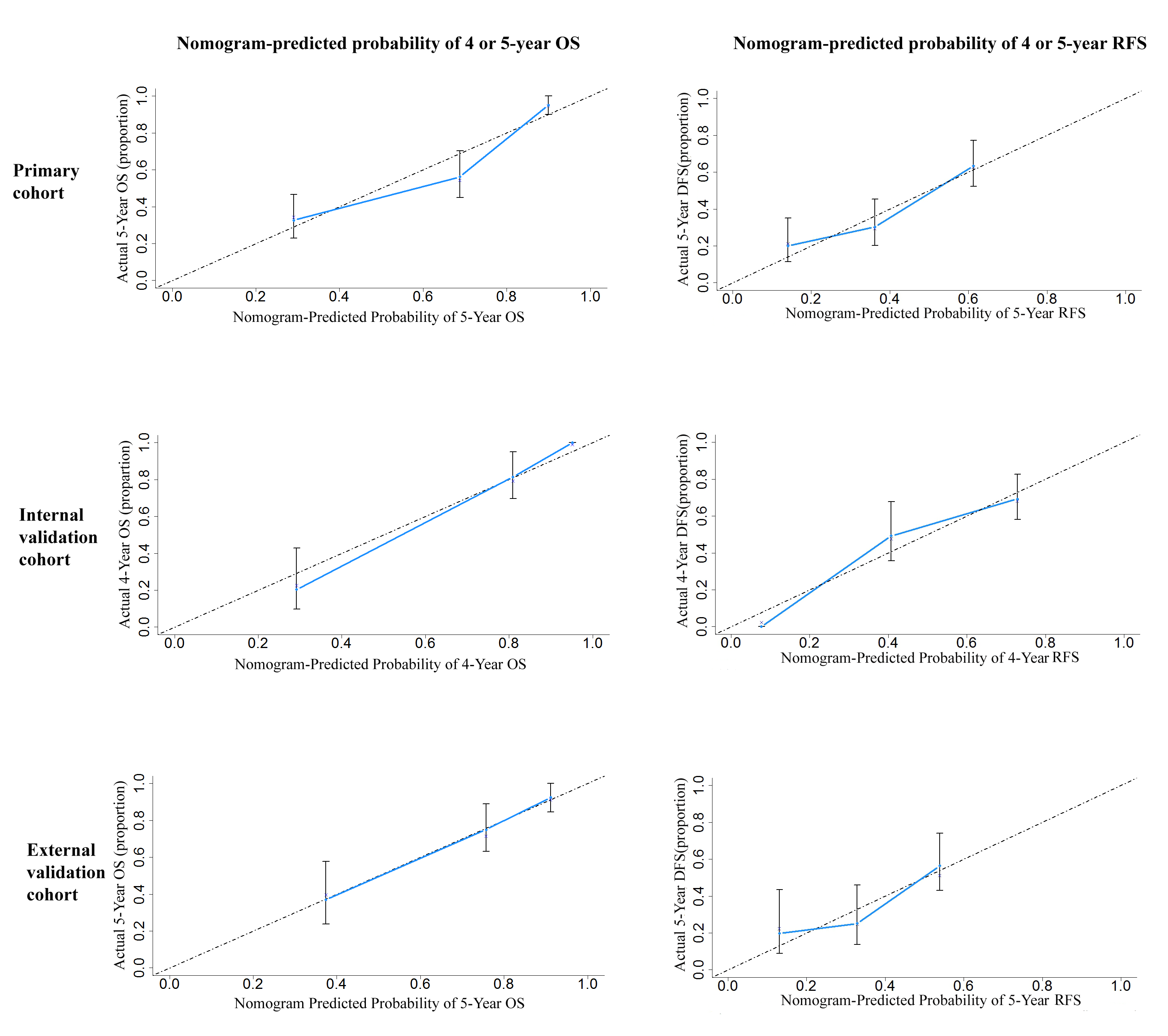

Supplement: Supplementary file 1 — Figure S1. The calibration curves for predicting the 4‐ or 5‐year overall survival (OS, A, C and E) and recurrence‐free survival (RFS, B, D and F) rates by nomogram prediction and actual observation in patients with hepatocellular carcinoma in the primary (A and B), internal validation (C and D) and external validation cohorts (E and F). [file CAM4-7-1170-s001.tif]

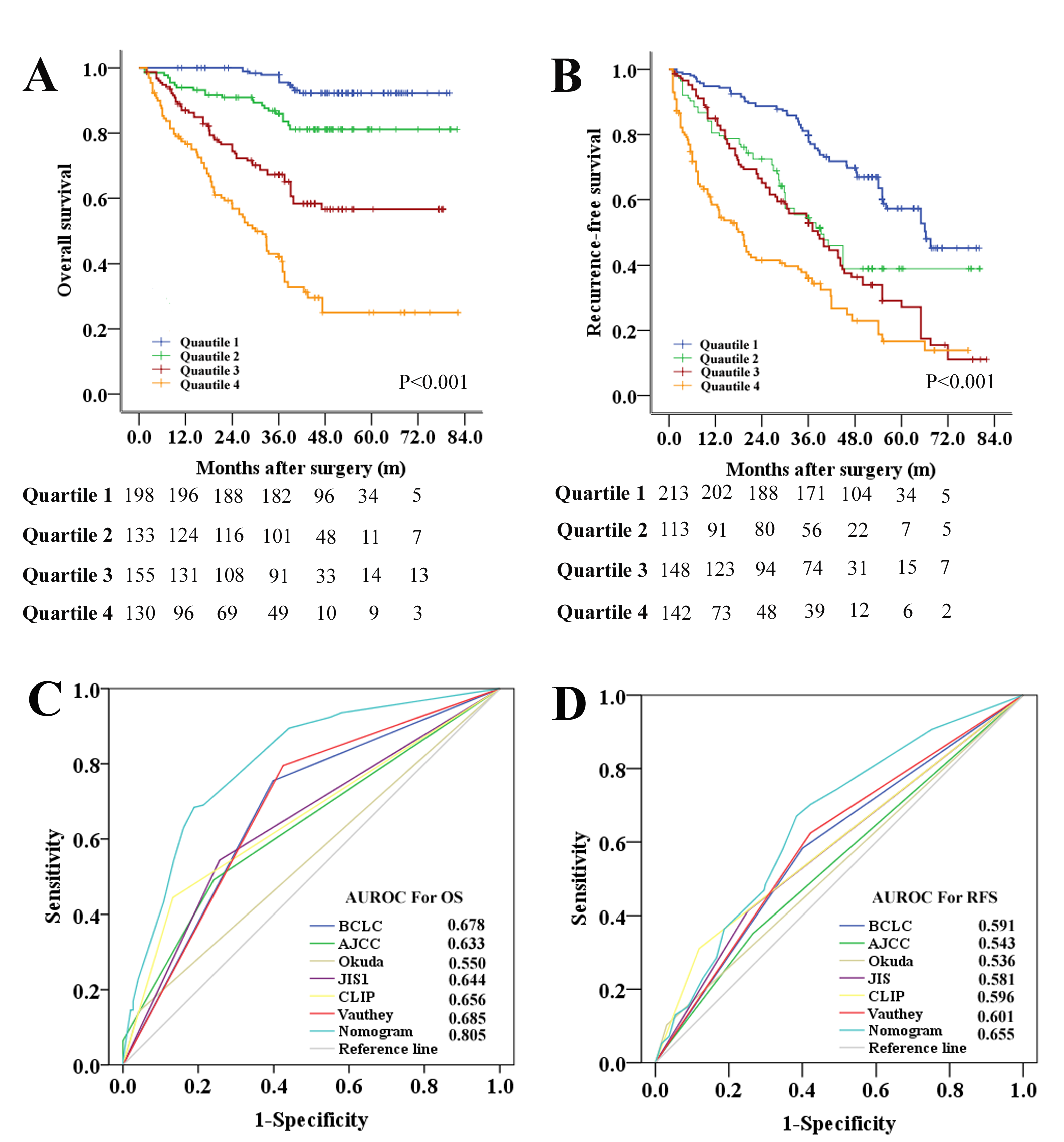

Supplement: Supplementary file 2 — Figure S2. Kaplan‐Meier survival curves and patients at risk at each year according to the quartiles of the overall survival (OS, A) and recurrence‐free survival (RFS, B) nomograms and predictive accuracy comparison between the OS (C) and RFS (D) nomograms and six conventional clinical staging systems by ROC curve analyses in the primary cohort. [file CAM4-7-1170-s002.tif]

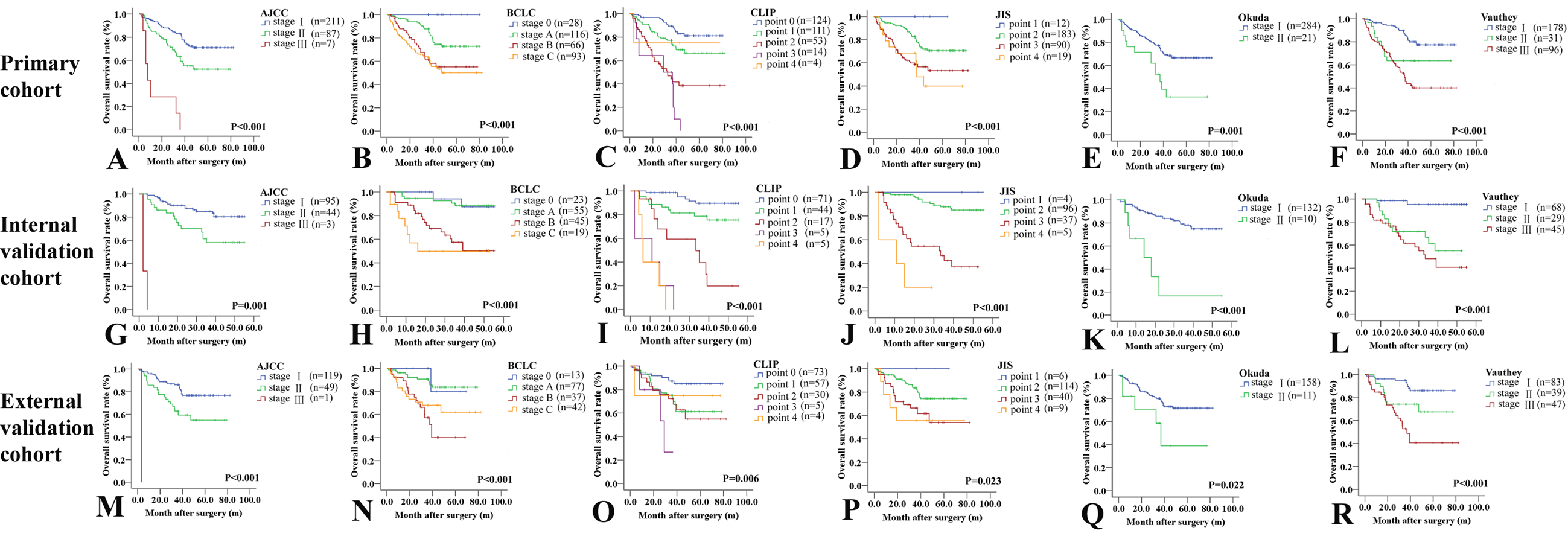

Supplement: Supplementary file 3 — Figure S3. Kaplan‐Meier survival curves of overall survival in the primary (A–F), internal validation (G–L) and external validation cohorts (M–R). [file CAM4-7-1170-s003.tif]

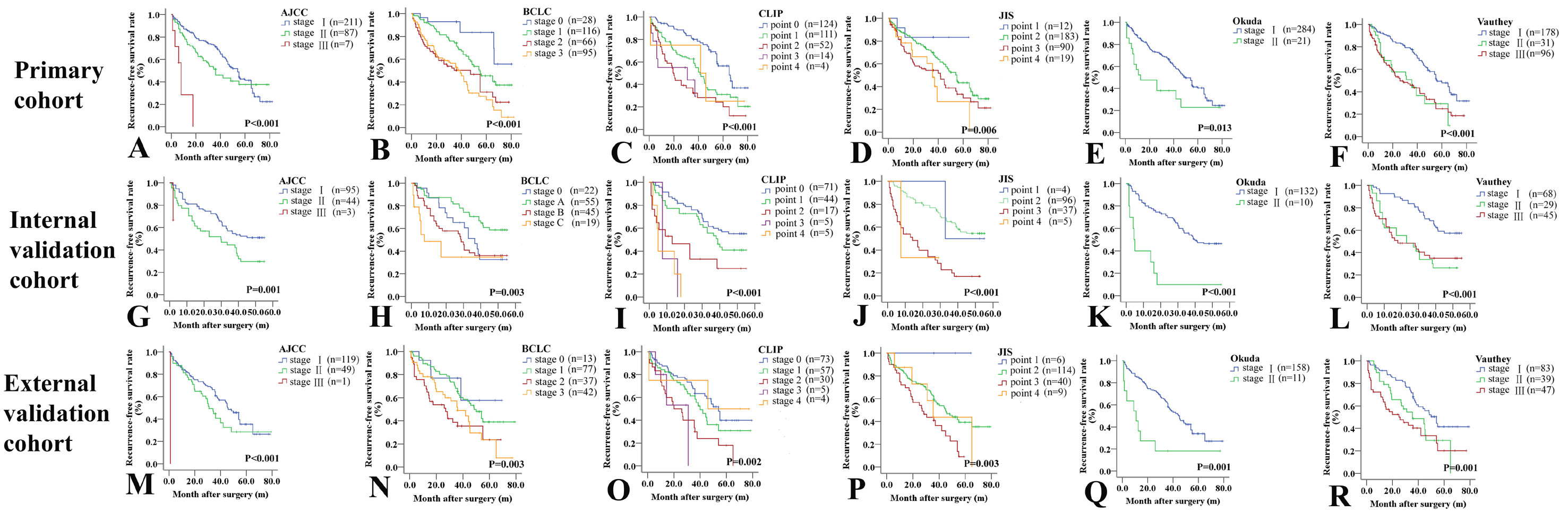

Supplement: Supplementary file 4 — Figure S4. Kaplan‐Meier survival curves of the recurrence‐free survival in the primary (A–F), internal validation (G–L) and external validation cohorts (M–R). [file CAM4-7-1170-s004.tif]

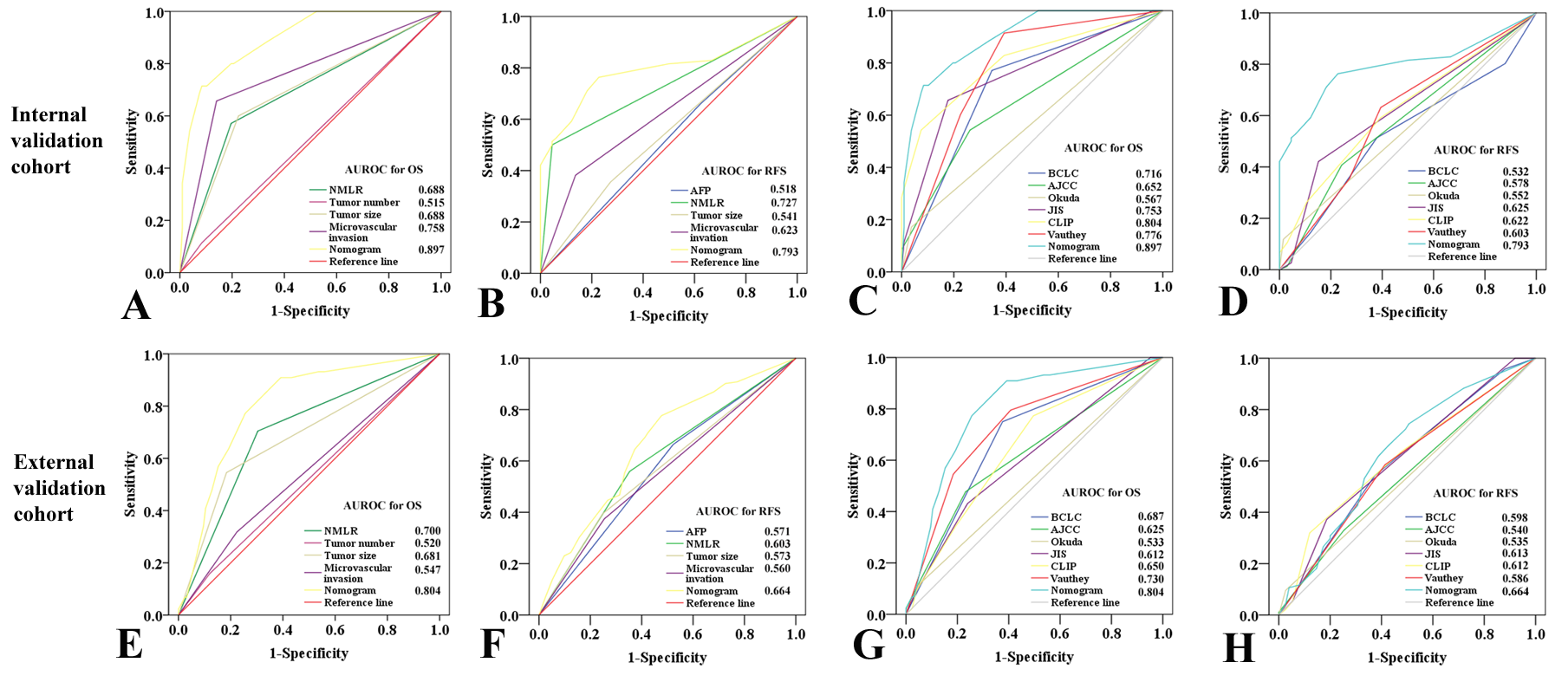

Supplement: Supplementary file 5 — Figure S5. Predictive accuracy comparison of each variable included in the OS (A and E) and RFS (B and F) nomograms and comparison between the OS (C and G) and RFS (D and G) nomograms and six conventional clinical staging systems by ROC curve analyses in the internal validation (A–D) and external validation (E–H) cohorts. [file CAM4-7-1170-s005.tif]

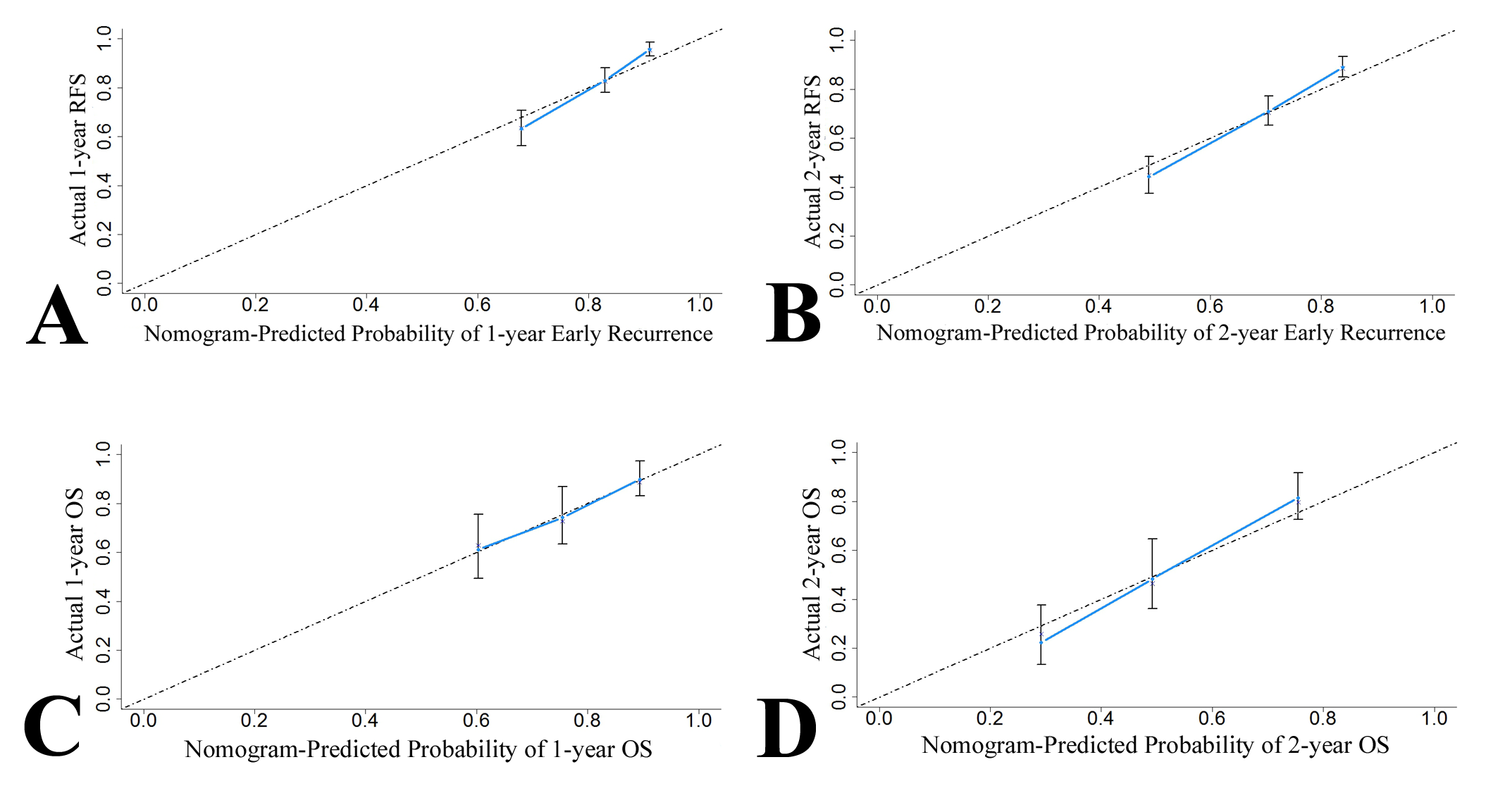

Supplement: Supplementary file 6 — Figure S6. The calibration curves for predicting the 1‐ and 2‐year early recurrence (ER, A and B) in the 616 patients with hepatocellular carcinoma and overall survival (OS, C and D) in the population with ER. [file CAM4-7-1170-s006.tif]
