# Supplementary material for: Comparison and validation of the prognostic value of preoperative systemic immune cells in hepatocellular carcinoma after curative hepatectomy
Source: Cancer Med. 2018 Mar 13;7(4):1170–82. doi: 10.1002/cam4.1424 (PMC5911633; doi:10.1002/cam4.1424)
Supplement: Supplementary file 7 — Table S1. Univariate analysis of overall survival and recurrence‐free survival of HCC in primary cohort. [file CAM4-7-1170-s007.docx]

**Table S1** **Univariate Analysis of Overall Survival and Recurrence-Free Survival of HCC in primary cohort**

| **Prognostic variables** | **OS** | | **RFS** | |
| --- | --- | --- | --- | --- |
|  | **HR(95%CI)** | **P-value** | **HR(95%CI)** | **P-value** |
| Age | 1.174 (0.778-1.772) | 0.445 | 1.370 (0.993-1.888) | 0.052 |
| Gender (Female/Male) | 0.836 (0.488-1.434) | 0.515 | 1.076 (0.689-1.681) | 0.745 |
| Cirrhosis (yes/no) | 0.636 (0.371-1.090) | 0.097 | 0.767 (0.484-1.218) | 0.257 |
| ALT | 1.150 (0.764-1.731) | 0.503 | 1.371 (0.994-1.892) | 0.052 |
| AST | 1.461 (0.967-2.207) | 0.070 | 1.395 (1.013-1.922) | **0.039** |
| GGT | 2.078 (1.359-3.178) | **0.001** | 1.608 (1.163-2.222) | **0.004** |
| ALB | 0.576 (0.377-0.881) | **0.010** | 0.762 (0.552-1.052) | 0.096 |
| TBIL | 0.773 (0.513-1.165) | 0.216 | 0.779 (0.566-1.073) | 0.123 |
| AFP (>20/≤20 ng/ml) | 1.556 (1.002-2.417) | **0.047** | 1.657 (1.183-2.322) | **0.003** |
| Neutrophil (>3.6/≤3.6x10^9^/L) | 1.005 (0.668-1.513) | 0.981 | 1.036 (0.754-1.424) | 0.827 |
| Monocyte (>0.4/≤0.4x10^9^/L) | 1.540 (1.022-2.320) | **0.037** | 1.491 (1.084-2.051) | **0.013** |
| Lymphocyte (>1.5/≤1.5x10^9^/L) | 0.448 (0.285-0.702) | **<0.001** | 0.711 (0.514-0.983) | **0.037** |
| Platelet (>144.0/≤144.0x10^9^/L) | 0.869 (0.577-1.309) | 0.502 | 0.767 (0.484-1.218) | 0.830 |
| HBsAg (Positive/Negative) | 1.616 (0.826-2.780) | 0.177 | 1.437 (0.933-2.212) | 0.098 |
| Tumor number (single/multiple) | 1.785 (1.066-2.989) | **0.025** | 1.522 (0.990-2.339) | 0.052 |
| Vascular invasion (yes/no) | 2.133 (1.413-3.219) | **<0.001** | 1.831 (1.315-2.548) | **<0.001** |
| Tumor capsule **(**omplete/Inomplete) | 1.180 (0.784-1.778) | 0.426 | 1.267 (0.921-1.741) | 0.142 |
| Tumor differentiation (Ⅰ-Ⅱ/Ⅲ-Ⅳ) | 1.439 (0.919-2.256) | 0.110 | 1.343 (0.946-1.908) | 0.096 |
| Tumor size (>5.0/≤5.0 cm) | 3.569 (2.360-5.396) | **<0.001** | 2.095 (1.511-2.905) | **<0.001** |
| NLR (>3.4/≤3.4) | 2.468 (1.633-3.730) | **<0.001** | 1.846 (1.308-2.606) | **<0.001** |
| MLR (>0.5/≤0.5) | 2.241 (1.466-3.426) | **<0.001** | 1.993 (1.382-3.874) | **<0.001** |
| PLR (>142.5/≤142.5) | 2.044 (1.334-3.133) | **0.001** | 1.590 (1.118-2.261) | **0.005** |
| PNLR (>480/≤480) | 1.435 (0.947-2.173) | 0.086 | 1.466 (1.062-2.024) | **0.019** |
| PMLR (>70.5/≤70.5) | 2.083 (1.359-3.194) | **0.001** | 1.789 (1.255-2.549) | **0.001** |
| NMLRv(>1.2/≤1.2) | 5.483 (3.364-8.934) | **<0.001** | 2.500 (1.808-3.457) | **<0.001** |

Abbreviations: OS: overall survival; RFS: recurrence-free survival; HCC: hepatocellular carcinoma; HR: hazard ratio; CI: confidence interval; ALT: alanine aminotransferase; AST: aspartate aminotransferase; GGT; gamma-glutamyl transpeptidase; ALB: albumin; TBIL: total bilirubin; AFP: alpha-fetoprotein; HBsAg: hepatitis B virus surface antigen.
