# Supplementary material for: Comparison and validation of the prognostic value of preoperative systemic immune cells in hepatocellular carcinoma after curative hepatectomy
Source: Cancer Med. 2018 Mar 13;7(4):1170–82. doi: 10.1002/cam4.1424 (PMC5911633; doi:10.1002/cam4.1424)
Supplement: Supplementary file 8 — Table S2. The C‐index of the predictors in nomograms and clinical staging systems. [file CAM4-7-1170-s008.docx]

**Table S2 The C-index of the predictors in nomograms and** clinical staging systems

| **Predictor** | **Overall survival (OS)** | | **Recurrence-free survival (RFS)** | |
| --- | --- | --- | --- | --- |
|  | **C-index** | **95% CI** | **C-index** | **95% CI** |
| Nomogram | 0.797 | 0.836-0.758 | 0.705 | 0.667-0.743 |
| **Independent prognostic factors** | | | | |
| NMLR | 0.698 | 0.654-0.742 | 0.633 | 0.592-0.674 |
| Tumor size | 0.659 | 0.609-0.709 | 0.606 | 0.565-0.647 |
| Vascular invasion | 0.598 | 0.546-0.650 | 0.563 | 0.522-0.597 |
| AFP | 0.540 | 0.490-0.590 | 0.572 | 0.531-0.613 |
| Tumor number | 0.538 | 0.538-0.578 | 0.527 | 0.496-0.558 |
| Clinical staging systems | | | | |
| CLIP score | 0.695 | 0.644-0.746 | 0.657 | 0.614-0.700 |
| Vauthey stage | 0.694 | 0.646-0.742 | 0.635 | 0.592-0.678 |
| BCLC stage | 0.670 | 0.621-0.719 | 0.626 | 0.583-0.669 |
| JIS score | 0.637 | 0.587-0.687 | 0.574 | 0.531-0.617 |
| AJCC stage | 0.622 | 0.568-0.676 | 0.577 | 0.534-0.620 |
| Okuda stage | 0.540 | 0.507-0.573 | 0.539 | 0.510-0.568 |

Abbreviations: NMLR: neutrophil and monocyte to lymphocyte ratio; AFP: alpha fetoprotein; CLIP: The Cancer of the Liver Italian Program; BCLC: Barcelona Clinic Liver Cancer; JIS: Japan Intergrated Staging; AJCC: American Joint Committee on Cancer.
